# Supplementary material for: Genotype and Phenotype Landscape of 283 Japanese Patients with Tuberous Sclerosis Complex
Source: Int J Mol Sci. 2022 Sep 22;23(19):11175. doi: 10.3390/ijms231911175 (PMC9569560; doi:10.3390/ijms231911175)
Supplement: Supplementary file 1 [file ijms-23-11175-s001.zip › Table S2.pdf]

Supplemental Table S2. Difference in each symptom and lesion between gender

Subject	All age (225)			Age ≥ 10 yr. (99)		
Sex (n)	Male (114)	Female (111)		Male (40)	Female (59)	
Lesion	n (%)	n (%)	p^*	n (%)	n (%)	p^*
Epilepsy All	114 (65.8)	111 (62.2)	0.5817	40 (72.5)	59 (54.2)	0.0920
Epilepsy S3	114 (56.1)	111 (49.5)	0.3513	40 (65.0)	59 (45.8)	0.0679
DD/ID All	84 (50.0)	98 (34.7)	0.0498	40 (47.5)	59 (33.9)	0.2107
DD/ID S3	84 (20.2)	98 (12.2)	0.1590	40 (25.0)	59 (8.5)	0.0429
Cort. Tuber	102 (78.4)	100 (69.0)	0.1508	34 (79.4)	50 (58.0)	0.0589
SEN	110 (73.6)	102 (80.4)	0.2583	37 (70.3)	52 (71.2)	1.0000
SEGA	108 (16.7)	104 (9.6)	0.1571	36 (22.2)	53 (9.4)	0.1278
Ret. Ham.	82 (17.1)	79 (22.8)	0.4312	25 (20.0)	39 (15.4)	0.7383
HM	113 (69.9)	110 (59.1)	0.0954	40 (67.5)	58 (53.4)	0.2107
AF	113 (37.2)	109 (45.9)	0.2205	40 (77.5)	58 (67.2)	0.3635
SP	111 (17.1)	106 (17.9)	1.0000	39 (35.9)	54 (29.6)	0.6536
UF	110 (10.9)	106 (19.8)	0.0885	39 (30.8)	54 (38.9)	0.5117
FP	111 (8.1)	108 (12.0)	0.3746	40 (15.0)	56 (17.9)	0.7867
Card. Rhabd.	102 (55.9)	88 (44.3)	0.1455	33 (45.5)	39 (15.4)	0.0086
Renal AML All	105 (20.0)	102 (36.3)	0.0129	38 (47.4)	57 (59.6)	0.2944
Renal AML S3	105 (3.8)	102 (12.7)	0.0229	38 (10.5)	57 (22.8)	0.1739
Renal Cyst	103 (18.4)	98 (28.6)	0.0981	38 (26.3)	54 (40.7)	0.1856
Renal Cyst S2+3	103 (9.7)	98 (21.4)	0.0306	38 (13.2)	54 (31.5)	0.0499
Liver AML	98 (4.1)	94 (9.6)	0.1575	35 (11.4)	53 (17.0)	0.5520
LAM	63 (0.0)	85 (20.0)	0.0000	26 (0.0)	54 (31.5)	0.0008
MMPH	31 (6.5)	38 (13.2)	0.4464	15 (13.3)	23 (21.7)	0.6807
Mutated gene						
TSC1	28 (24.6)	28 (25.2)		12 (30.0)	21 (35.6)	
TSC1/2		1 (0.9)			1 (1.7)	
TSC2	62 (54.4)	55 (49.5)		21 (52.5)	20 (33.9)	
NMD	24 (21.1)	27 (24.3)		7 (17.5)	17 (28.8)	
Age distribution						
Age range (yr.)	0-56	0-80		10-56	10-80	
Average age (yr.)	10.14	20.13		24.8	35.86	
Median age (yr.)	4	13		21	33	

Gender differences were analyzed in clinically definite TSC patients (n = 255). *Fisher's exact test; S3: score 3; S2+3: score 2 or 3 in Table 13. AF: facial angiofibroma; AML: angiomyolipoma; Card. Rhabd.: cardiac rhabdomyoma; Cort.Tuber: cortical tubers; DD/ID: developmental delay/intellectual disability; FP: forehead plaque; HM: hypomelanotic macules; LAM: lymphangiomyomatosis; MMPH: Multifocal micronodular pneumocyte hyperplasia; NMD: no mutation detected; Ret. Ham.: retinal hamartoma; SEGA: subependymal giant cell astrocytoma; SEN: subependymal nodules; SP: shagreen patch; UF: ungual fibroma.
